# Complete Genome Sequence of the Ebosin-Producing Strain *Streptomyces* sp. 139

**DOI:** 10.1128/MRA.01283-19

**Published:** 2019-12-05

**Authors:** Limei Ai, Mengxin Geng, Ming Ma, Liping Bai

**Affiliations:** a NHC Key Laboratory of Biotechnology of Antibiotics, Institute of Medicinal Biotechnology, Chinese Academy of Medical Sciences & Peking Union Medical College, Beijing, China; b CAMS Key Laboratory of Synthetic Biology for Drug Innovation, Institute of Medicinal Biotechnology, Chinese Academy of Medical Sciences & Peking Union Medical College, Beijing, China; Portland State University

## Abstract

Members of the genus Streptomyces are known for their ability to produce compounds with various bioactivities and for their complex morphologies. *Streptomyces* sp. strain 139 is the producer strain of the exopolysaccharide (EPS) ebosin, which has remarkable *in vivo* antirheumatic arthritis activity. Here, we report its complete genome sequence, which will facilitate the study of the biosynthesis of ebosin.

## ANNOUNCEMENT

Our lab previously reported the isolation, biosynthesis, and bioactivity of ebosin from Streptomyces sp. strain 139 (1–3). Ebosin belongs to the exopolysaccharide (EPS) family and has been demonstrated to have anti-rheumatic arthritis activity *in vivo* and antagonist activity for interleukin I receptor (IL-1R) *in vitro* (1, 2).

Here, we report the genome sequence of *Streptomyces* sp. 139, including the full sequence of two megaplasmids and a chromosomal sequence. The sequence will be of interest for future studies aimed at identifying novel bacterial metabolites with various bioactivities and for further characterization of the biosynthesis of ebosin.

*Streptomyces* sp. 139 was cultured in 10 ml tryptic soy broth (TSB) medium (3%; Difco) at 28°C for 36 h. The culture was then inoculated 1:10 (vol/vol) into 50 ml fresh TSB medium and incubated at 28°C for 24 h. After centrifugation at 5,000 rpm for 10 min, the cell pellet was harvested, flash frozen in liquid nitrogen, and sent out for genomic DNA extraction (Beijing Genomics Institute, Shenzhen, China). Genomic DNA from *Streptomyces* sp. 139 was isolated using the cetyltrimethylammonium bromide (CTAB) protocol as previously described (4).

According to the report provided by the Beijing Genomics Institute, the Illumina HiSeq library was constructed using the Illumina Nextera DNA Flex kit, and the PacBio SMRTbell library was prepared using the SMRTbell template prep kit, following the manufacturer’s instructions. Whole-genome sequencing was performed using the PacBio RS II and Illumina HiSeq 4000 platforms with a paired-end sequencing strategy (Beijing Genomics Institute, Shenzhen, China). The Illumina sequencing library has a mean read length of 125 bp and 9,560,136 total reads. The PacBio sequencing library has a subread mean length of 8,898 bp and 57,850 total reads. The draft genomic contig, which was a group of fragments following self-correction with pbdagcon (https://github.com/PacificBiosciences/pbdagcon), was assembled using Celera Assembler 8.3 (https://www.mybiosoftware.com/celera-assembler-6-1-genome-shotgun-assembler.html). To improve the accuracy of the genome sequence, GATK (https://www.broadinstitute.org/gatk/) and SOAP tool packages, including SOAP2, SOAPsnp, and SOAPindel (5), were used to make single-base corrections. The presence of two plasmids was identified by mapping the Illumina reads to the bacterial plasmid database (http://www.ebi.ac.uk/genomes/plasmid.html). Default parameters were used for all software. The resulting genome sequence has a 70.0× coverage depth. The draft genome has 3 contigs, an *N*_50_ value of 19,755 bp, and an approximate size of 7,343,456 bp, with a G+C content of 72.19%. Gene annotation was performed using the NCBI Prokaryotic Genome Annotation Pipeline (6).

### Data availability.

This draft genome sequence has been deposited at GenBank under accession no. CP043959 to CP043961. Contig CP043959 is the bacterial chromosome, whereas contigs CP043960 and CP043961 are two plasmids. The publicly available raw data are available under SRA accession no. PRJNA565833.
